# Supplementary figures and images for: 4DNvestigator: time series genomic data analysis toolbox
Source: Nucleus. 2021 Apr 2;12(1):58–64. doi: 10.1080/19491034.2021.1910437 (PMC8049205; doi:10.1080/19491034.2021.1910437)

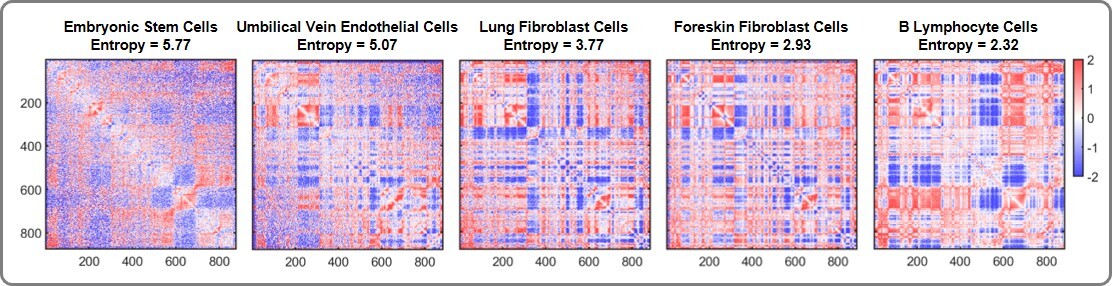

Supplement: Supplemental Material [file KNCL_A_1910437_SM5566.zip › Entropy_examples_v1.jpeg]

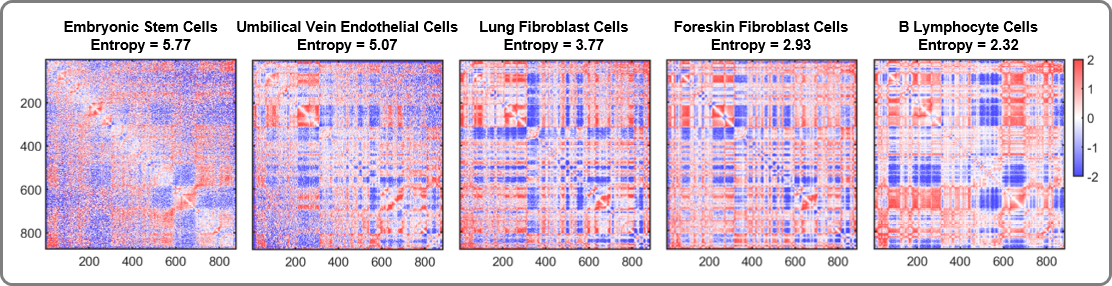

Supplement: Supplemental Material [file KNCL_A_1910437_SM5566.zip › Entropy_examples_v1.png]

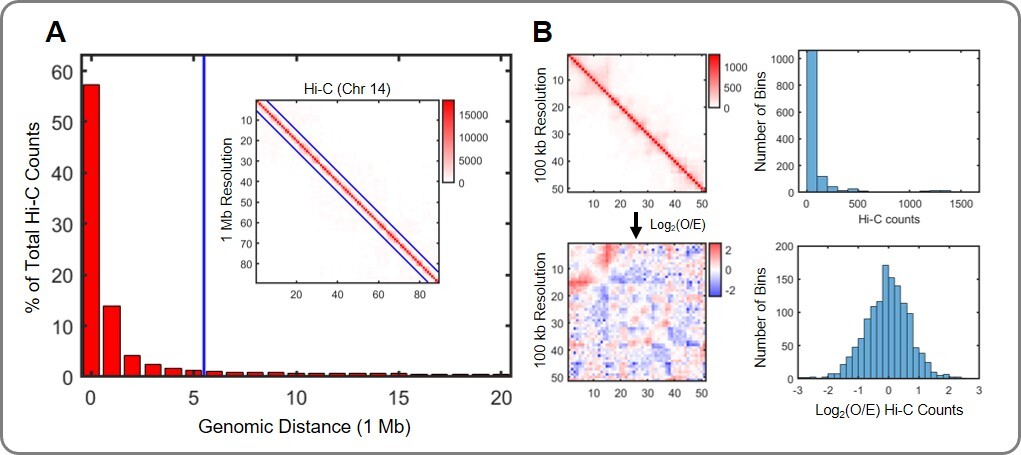

Supplement: Supplemental Material [file KNCL_A_1910437_SM5566.zip › hicNormalization_v1.jpeg]

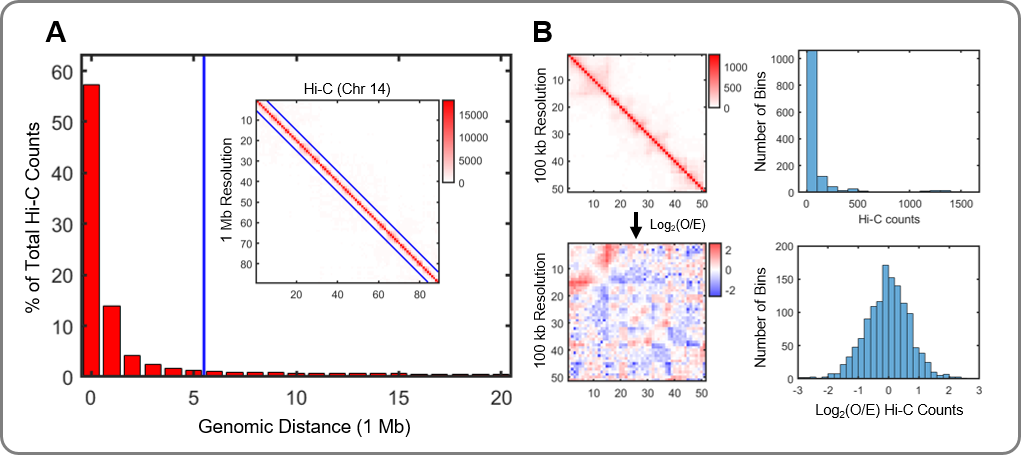

Supplement: Supplemental Material [file KNCL_A_1910437_SM5566.zip › hicNormalization_v1.png]

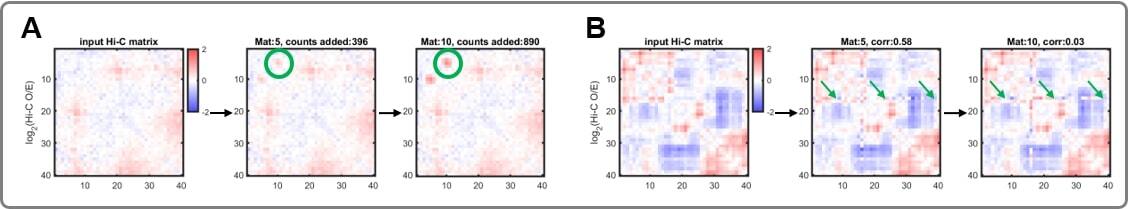

Supplement: Supplemental Material [file KNCL_A_1910437_SM5566.zip › lpBenchmarking7.jpeg]

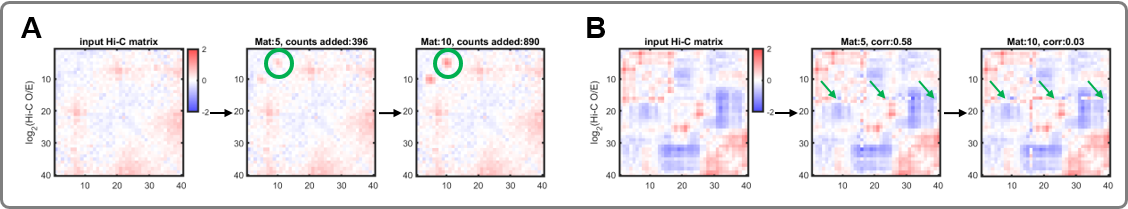

Supplement: Supplemental Material [file KNCL_A_1910437_SM5566.zip › lpBenchmarking7.png]

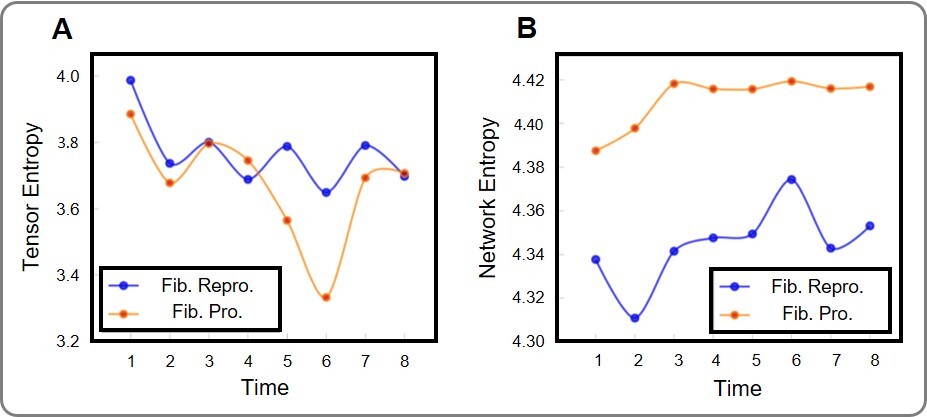

Supplement: Supplemental Material [file KNCL_A_1910437_SM5566.zip › tensorEntropy_v1.jpeg]

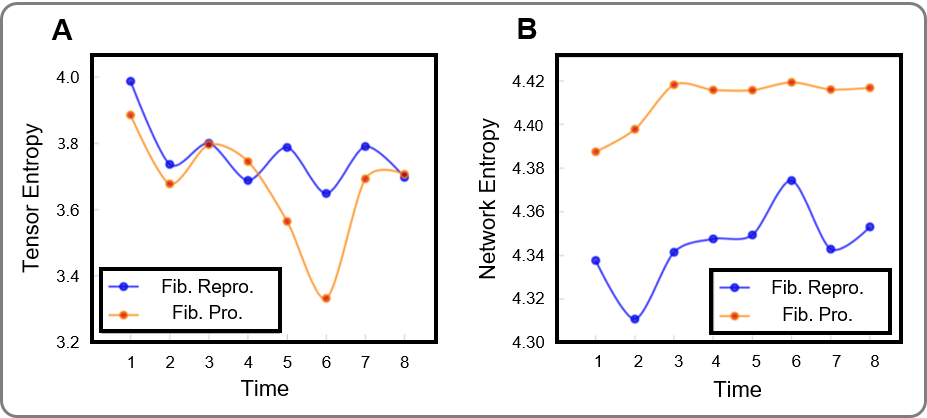

Supplement: Supplemental Material [file KNCL_A_1910437_SM5566.zip › tensorEntropy_v1.png]
